# Etiological analysis of 167 cases of drug-resistant epilepsy in children

**DOI:** 10.1186/s13052-024-01619-8

**Published:** 2024-03-13

**Authors:** Ran-Ran Zuo, Mei Jin, Su-Zhen Sun

**Affiliations:** 1 Department of Neurology, Hebei Childrens Hospital, 133, Jianhua South Street, 050000 Shijiazhuang, Hebei Province China; 2 The Key Laboratory of Pediatric Epilepsy and Neurological Disorders of Hebei Province, 050000 Shijiazhuang, Hebei China

**Keywords:** Drug-resistant epilepsy, Etiology, Children, Phenotype

## Abstract

**Background:**

To analyze the etiological distribution characteristics of drug-resistant epilepsy (DRE) in children, with the aim of providing valuable perspectives to enhance clinical practice.

**Methods:**

In this retrospective study, clinical data were collected on 167 children with DRE who were hospitalized between January 2020 and December 2022, including gender, age of onset, seizure types, video electroencephalogram(VEEG) recordings, neuroimaging, and genetic testing results. Based on the etiology of epilepsy, the enrolled children were categorized into different groups. The rank-sum test was conducted to compare the age of onset for different etiologies.

**Results:**

Of the 167 cases, 89 (53.3%) had a clear etiology. Among them, structural factors account for 23.4%, genetic factors for 19.2%, multiple factors for 7.2%, and immunological factors for 3.6%. The age of onset was significantly earlier in children with genetic causes than those with structural (*P* < 0.001) or immunological (*P* = 0.001) causes.

**Conclusions:**

More than half of children with DRE have a distinct underlying cause, predominantly attributed to structural factors, followed by genetic factors. Genetic etiology primarily manifests at an early age, especially among children aged less than one year. This underscores the need for proactive enhancements in genetic testing to unveil the underlying causes and subsequently guide treatment protocols.

**Supplementary Information:**

The online version contains supplementary material available at 10.1186/s13052-024-01619-8.

## Introduction

Epilepsy is a prevalent chronic neurological disorder characterized by recurrent, episodic, and transient neurological dysfunctions resulting from excessive neuronal discharges [1]. Most people with epilepsy are able to achieve seizure freedom through regular intake of antiseizure medications (ASMs). However, approximately one-third of pediatric patients still experience uncontrollable seizures after receiving two or more antiepileptic medications, a condition known as drug-resistant epilepsy (DRE) [2]. For children with DRE, not only is the mortality rate significantly high, but it also profoundly impacts their quality of life [3]. A pronounced correlation exists between the frequency of seizures and the levels of anxiety or depression. Patients with DRE often experience increased anxiety or depression, and conversely, elevated anxiety and depression can worsen seizure frequency and intensity. The prevalence of psychiatric disorders among individuals with epilepsy is tenfold higher than that in the general population [4].

In recent years, despite the continuous introduction of new medications, the treatment status of DRE has seen little significant improvement compared to the past two decades [5]. According to reports [6–8], some non-pharmacological therapies (such as epilepsy surgery, ketogenic diet, and neurostimulation techniques ) have reduced seizures for some DRE patients. However, due to the complexity of epileptic episodes, the heterogeneity of epilepsy syndromes and the presence of comorbidities, determining an appropriate treatment regimen for DRE remains challenging. Etiology, as a key prognostic factor, closely correlates with clinical phenotypes and can play a vital role in treatment decisions. In this study, we have attempted to summarize the clinical features and etiological distribution of children with DRE. Additionally, we have conducted a comparative examination of the age of onset related to different etiological factors, aiming to provide clinical guidance.

## Methods

### Study population

This study included children admitted to the Neurology Department of Hebei Children’s Hospital from January 2020 to December 2022, meeting the diagnostic criteria for DRE as proposed by International League against Epilepsy (ILAE) [9]. The exclusion criteria are as follows: (1) Pseudo-refractory epileptic events caused by medically induced epilepsy resulting from irrational medication usage, inadequate adherence to prescribed treatment by both the guardian and child; (2) Individuals with underlying conditions such as congenital heart disease or blood disorders; (3)

Individuals with incomplete clinical data. This study has been approved by the Ethics committee of Children’s Hospital Affiliated to Hebei Medical University (202,103). All guardians/parents gave written informed consent in accordance with the Helsinki Declaration of 1975.

In accordance with the diagnostic criteria for DRE proposed by ILAE in 2010 [9], seizure-free duration does not exceed three times the pre-treatment maximum seizure interval or one year (whichever is longer) after rational selection and correct use of at least two well-tolerated antiepileptic drugs alone or in combination, depending on the type of seizure.

The definition and classification of epilepsy syndromes adhere to the 2022 ILAE position paper [10]. It refers to a group of epileptic disorders characterized by distinctive clinical and electroencephalographic phenotypes, typically associated with specific etiologies (structural, genetic, metabolic, immunological, and infectious causes). Based on the age of onset, it is categorized into neonatal and infantile onset, childhood onset, onset at any age, and idiopathic generalized epilepsy. According to the seizure types and classification of epilepsy established by ILAE in 2017, seizure types are categorized into focal seizures, generalized seizures, and seizures of unknown origin [11]. The seizure types of enrolled children are assessed by specialized pediatric neurologists based on descriptions provided by guardians or video recordings, in conjunction with video electroencephalogram results.

### Data collection and grouping

Data on DRE, including gender, age of onset, seizure types, medication categories, VEEG recordings, neuroimaging findings, genetic test findings, blood and urine genetic metabolic screening and neuroimmunological analyses were collected.

Peripheral blood samples were collected from both patients and their family members to extract DNA. Whole-exome sequencing was employed for gene variant screening, followed by Sanger sequencing to verify suspected pathogenic variants and their origins. Some children underwent chromosomal karyotyping, copy number variation detection(CNV), and mitochondrial gene testing. The pathogenicity of genetic variants was assessed according to the classification standards established by the American College of Medical Genetics and Genomics (ACMG) [12].

All the enrolled children regularly completed the 15-hour night VEEG examination at Hebei Children’s Hospital, and the results were evaluated by professional brain electrophysiology experts. 3.0T cranial MRI is employed to evaluate the presence of epilepsy-related structural abnormalities in the children. The imaging results undergo joint evaluation by radiology and neurology experts.

Based on the age of seizure onset, these patients have been categorized into groups of < 1 year, 1–3 years, 3–6 years, and ≥ 6 years. According to the 2017 ILAE classification of epilepsy etiology [13], they have been divided into structural, genetic, metabolic, infectious, immunological, and unknown etiology groups. If a patient has two or more etiologies, such as genetic and structural causes, it is classified as having multiple etiologies.

### Statistical analysis

The data were analyzed using SPSS26.0 statistical software. Non-normally distributed measurements were presented as median (interquartile range) [M(Q1, Q3)], and comparisons between groups were conducted using the rank-sum test. Count data were reported as the number of cases and percentage (%). Statistical significance was defined as *P* < 0.05.

## Results

### Characteristics of the study population

The study included 167 children with DRE, 100 of them were males (59.9%), the median age of onset was 3 (0.5, 5) years and the median number of antiepileptic drugs used was 3 (2, 4). With regard to the age of onset, there were 56 instances observed in children younger than one year, 26 cases occurring between the ages of 1 to 3 years, 49 cases between 3 and 6 years, and 36 cases among those aged six years or older. 60 cases (35.9%) were diagnosed with epileptic syndromes or epileptic encephalopathies, while 107 cases (64.1%) were diagnosed with non-syndromic epilepsies. A comprehensive exposition of the specific clinical manifestations can be found in Table 1.

**Table 1:** Clinical manifestations of DRE

Clinical manifestations	Patients, No.(%)
*Epilepsy syndromes*	60 (35.9)
**Epilepsy syndromes with onset in neonates and infants**	36 (60)
Infantile epileptic spasm syndrome (IESS)	14
Dravet syndromes	6
Etiology specific epileptic encephalopathy	4
Early-infantile DEE (EIDEE)	2
Lennox-Gastaut syndrome	1
Hard to classification of epilepsy syndrome	9
**epilepsy syndromes with onset in childhood**	24 (40)
Developmental and/or epileptic encephalopathies (DEEs)	17
Lennox-Gastaut syndrome	4
Febrile infection-related epilepsy syndrome,FIRES	2
Hemiconvulsion–hemiplegia–epilepsy syndrome, HHE	1
Hard to classification of epilepsy syndrome	10
Self-limited focal epilepsies (SeLFEs)	7
*Others*	107 (64.1)
**Focal seizures**	53 (49.5)
**Generalized seizures**	16(14.9)
Generalized tonic-clonic seizure,GTCS	11
Absence seizures	3
Clonic seizures	1
Myoclonic seizures	1
**Multiple seizure types**	40(37.3)

### Results of genetic testing

**Fig. 1:**
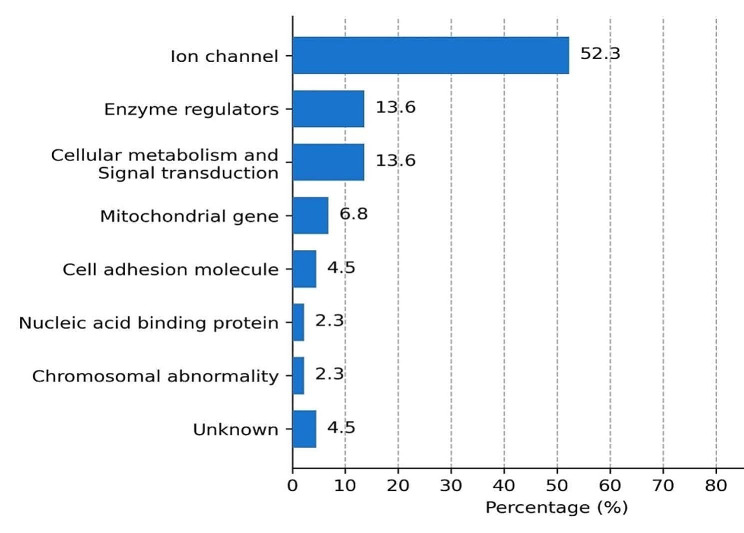
The proportion of different functional genes

A total of 95 children (56.9%) underwent genetic assessment. Among them, 44 children (46.3%) exhibited abnormal results from the genetic analysis, with 12 cases showing combined structural or metabolic abnormalities classified as multiple etiologies. In sum, there were 30 gene mutations identified, including 29 single-gene mutations and one chromosomal abnormality. These mutated genes were categorized based on the functions of the encoded proteins, as detailed in Table 2. It is noteworthy that genes associated with the ion channel class constituted a substantial portion of the cases, amounting to 52.3% (Fig. 1).

**Table 2:** The gene codes for protein function

Code for protein function	Functional subclassification	Gene related to epilepsy(OMIM )
Ion channel	Voltage-gated sodium channel (Nav)	SCN1A, SCN2A, SCN8A
	Voltage-gated potassium channel (Kv)	KCNQ2, KCNT1, KCNB1
	Voltage-Gated Calcium Channel (Cav)	CACNA1A, CACNA1E
	Voltage-Gated chloride Channel (CIC)	CLCN4
	Ligand-gated ion channel GABA receptors	GABRA1
	Ligand-gated ion channels glutamate receptors	GRIN2B
Enzyme regulators		CDKL5, CHD2, OTC, PAH, PCK1, PIGA
Cellular metabolism and Signal		FGF12, DEPDC5, TSC1, TSC2
Cell adhesion molecule (CAM)		L1CAM, PCDH19
Nucleic acid binding protein (NABPS)		SMC1A
Mitochondrial gene		DNM1L, MT-TL1, POLG
Chromosomal abnormality		Chromosomal 18q deletion syndrome
Unknown		RANBP2, STAMBP

### Etiological distribution and correlation with age of onset of epilepsy

**Fig. 2:**
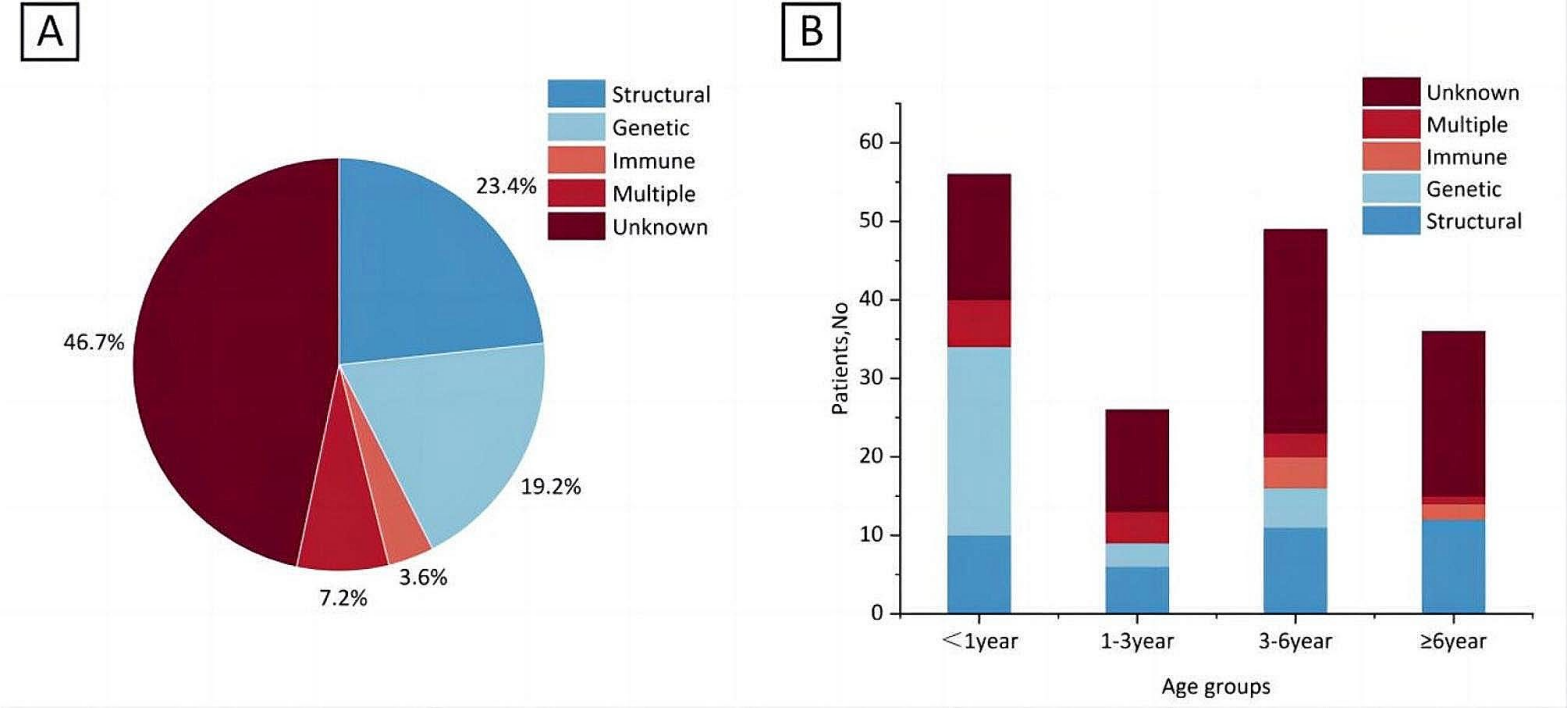
**A**, Etiological distribution of DRE; **B**, The bar chart shows the differences in etiology distribution among age groups

89 children (53.3%) had a definite etiology. Among them, 39 (23.4%) were attributed to a structural etiology, 32 (19.2%) were ascribed to a genetic etiology, 12 (7.2%) manifested multiple etiological factors, and 6 (3.6%) were associated with an immunological etiology (Fig. 2A). The distribution of causes varies across different age groups, with the highest proportion of genetic causes observed in infants under one year old. Conversely, structural causes constitute the largest proportion among individuals aged six years or older.(Fig. 2B). A significant distinction was evident in the age of onset among the diverse etiological categories (*P* < 0.001) (Table 3). Upon further comparative analysis, it was established that genetic etiologies presented with an earlier age of onset in comparison to both structural etiologies (H=-28.429, *P* < 0.001) and immunological etiologies (H = 43.333, *P* = 0.001).

**Table 3:** The rank sum test was used to compare the onset age of different causes

Etiology	Structural	Genetic	Multiple	Immune
Age of onset(year) *M(Q1, Q3)*	3(0.67, 6)*	0.46(0.19, 0.96)	1.29(0.5, 3)	4.5(3.79, 6.25)*
*H*	26.741			
*P*	< 0.001			

Note: *Compared to the genetic group, *P*<0.05

### Specific etiology and clinical manifestations

Structural etiology included congenital structural anomalies in 14 cases (35.9%) and acquired structural abnormalities in 25 cases (64.1%) (Table 4). The clinical manifestations comprised IESS in 4 cases, SeLECTS in 2 cases, FIRE in 1 case, unclassified epileptic encephalopathy in 4 cases, and non-syndromic cases in 28 instances (comprising focal seizures in 16 cases, generalized seizures in 1 case, absence of concentration seizures in 1 case, and multiple seizure types in 10 cases).

**Table 4:** Distribution of structural causes

Acquired structural abnormalities(25,64.1%)		Congenital structural abnormality(14,35.9%)	
Category	Number(%)	Category	Number(%)
Hypoxic ischemic encephalopathy or hypoglycemic brain damage	10(40.0%)	MCD	4(28.6%)
Encephalitis	9(36.0%)	Hippocampal abnormality	3(21.4%)
Trauma	2(8.0%)	Strurge-Weber	2(14.3%)
Purulent meningitis	2(8.0%)	Rasmussen encephalitis	1(7.1%)
Cerebral hemorrhage	1(4.0%)	Systemic sclerosis	1(7.1%)
Unknown	1(4.0%)	TSC	1(7.1%)
		Unknown	2(14.3%)

Note: MCD, malformations of cortical development; TSC, Tuberous sclerosis complex

The assemblage of 32 cases with genetic etiology encompassed 20 solitary gene mutations, comprised of 9 SCN1A, 2 KCNQ2, and one each of eight other distinct single gene mutations. Furthermore, one case featured a chromosomal abnormality in the form of the 18q deletion syndrome. 53% (17/32) manifested as epileptic syndromes. Among them, 5 cases of Dravet syndrome patients manifested SCN1A gene variations. The 8 cases of DEES showed variations in FGF12, SCN8A, KCNQ2, KCNB1, CACNA1A, CDKL5, CHD2, and STAMBP. In 3 cases of IESS, associations with SCN1A, CACNA1E, and PHGDH variations were observed, while 1 case of EIDEE was marked by a GABRA1 variation. Furthermore, 47% manifests as non-epileptic syndrome.This encompasses 9 cases of focal epileptic seizures, 3 instances of generalized seizures, and 3 occurrences of multiple seizure types. Among these non-epileptic syndromes, 3 cases are marked by SCN1A mutations, while the remaining 12 exhibit a singular gene mutation each.

Six children of immunological etiology exhibited psycho-behavioral alterations, including fear and the use of indecent language during disease onset. Among them, one case was diagnosed with NMDAR receptor encephalitis, and another case was confirmed as Rasmussen’s encephalitis. Two cases displayed elevated cerebrospinal fluid white cell counts and immunoglobulin levels, indicating an immune response within the central nervous system. In one patient, cerebrospinal fluid antibody testing targeting IgG oligoclonal bands was positive, while the remaining case showed no abnormalities in routine cerebrospinal fluid analysis, biochemistry, or neuronal antibodies. Following immunotherapy, the psycho-behavioral symptoms in these children demonstrated marked improvement, yet persistent epileptic seizures persisted, with suboptimal response to antiepileptic drug therapy. The clinical phenotypes were characterized by focal seizures in 3 instances and focal secondary generalized seizures in others.

In 8 out of the 12 multifactorial cases, both genetic and structural factors were in play. Among these, 4 cases featured mutations in the TSC gene, which were consistent with manifestations of tuberous sclerosis on cranial MRI. Additionally, 1 case each of KCNT1, CACNA1A, PCK1, and OTC variants manifested unilateral floppy foci, cerebral atrophy, or hippocampal sclerosis on cranial MRI. In 4 cases, both genetic and metabolic factors converged, comprising 2 cases of mitochondrial encephalomyopathy (POLG and MT-TL1 variants, respectively), 1 case with a DNM1L gene variant with clinical manifestations of mitochondrial peroxisome cleavage-deficient lethal encephalopathy type I(OMIM#614,388), and 1 case with a PAH gene variant and clinical manifestations of phenylketonuria with seizures. The clinical phenotypes encompassed LGS in 1 case, Dravet in 1 case, epileptic encephalopathy that defied classification in 2 cases, and non-syndromic in 10 cases, including focal seizures in 4 cases, generalized seizures in 2 cases, and multiple seizure types in 2 cases.

## Discussion

This study summarized the etiological distribution characteristics and corresponding clinical manifestations of 167 children with DRE, establishing a foundation for the precise diagnosis and treatment of pediatric DRE. 53.3% exhibited identifiable causes, predominantly structural, followed by genetic causes, with a minority having immunological or multifactorial etiologies. Onset ages varied among different causes, with genetic causes manifesting earlier than structural and immunological ones. Regarding clinical presentations, 60 cases displayed a specific epileptic syndrome, among which 36 cases (60%) had onset in the neonatal and infantile period. In the 107 cases of non-syndromic epileptic syndromes, 53 cases (49.5%) were characterized by focal epileptic seizures, and 40 cases (37.3%) presented with diverse seizure types.

The pathogenesis of DRE remains somewhat elusive to date. However, numerous studies have proposed several primary hypotheses through in vivo or in vitro experiments, including the drug transporter hypothesis, neural network hypothesis, drug target hypothesis, genetic mutation hypothesis, disease severity hypothesis, and pharmacokinetic hypothesis. Yet, these hypotheses have inherent limitations and areas where they overlap [14]. Presently, the role of neuroinflammation in the pathogenesis of DRE is gradually being elucidated. Neuroinflammation can result in compromised blood-brain barrier, neuronal loss, and excessive neuronal excitation, not only triggering epileptic seizures but also correlating with the occurrence of DRE [15]. Recent research has demonstrated that neuroinflammation could potentially serve as a common underlying mechanism among various hypotheses in DRE and might be a prospective target for its treatment [16]. Promising therapeutic effects and safety have been observed in DRE and status epilepticus with drugs targeting IL-1, IL-6, and CD20 [17]. However, clinical data on this front remains limited, warranting further research for validation.While the pathogenic mechanisms of DRE are still in the exploratory phase, the analysis of etiology can also offer instructive significance for therapeutic interventions.

Children with imaging abnormalities are more likely to develop DRE than children with normal imaging [18]. Malformations of Cortical Development (MCD) constitute the principal congenital structural anomalies. Acquiring structural cerebral abnormalities, primarily due to hypoxic-ischemic encephalopathy or hypoglycemia brain damage, is a major etiological factor in the onset of DRE during infancy [19]. Subsequently, residual foci of cerebral softening and glial proliferation following encephalitis represent another notable contributing factor. It has been documented that approximately 10% of pediatric encephalitis patients progress to DRE, with the herpes simplex virus (3/9, 33%) and unidentified (8/40, 20%) forms of encephalitis accounting for relatively common causative agents [20]. For children with structural lesions, it is imperative to embark on an early surgical evaluation, unhampered by considerations of age, coexisting medical conditions, or the type of seizures [18]. There exists compelling evidence supporting the supremacy of surgical intervention over pharmaceutical approaches in the treatment of DRE in children [8]. In cases where epilepsy is precipitated by a well-defined lesion within the nonverbal cortex, the contemplation of preoperative evaluation should be undertaken even before the onset of drug-resistant epilepsy [21].

The genetic etiology is closely linked to drug-resistant epilepsy, particularly in severe cases of DEES such as the majority of Dravet syndromes where resistance to medication is commonly observed [22]. Approximately 80% of Dravet syndrome cases are associated with SCN1A variation [23], which aligns with the findings of this study. Additionally, IESS patients are also commonly associated with genetic factors, and cognitive dysfunction and stagnation are among its clinical phenotypes [24]. Predominantly, genetic variations are attributed to ion channel genes, including but not limited to SCN1A and KCNQ2, echoing the observations of Liu et al [25, 26]. DRE related genes also include enzyme regulators,exemplified by CDKL5, elements of cellular metabolism and signal transduction, typified by FGF12, and cell adhesion molecules such as PCDH19. It is worth noting that in this study, a child with chromosome copy number variation was found to have 13.6 Mb deletion in the 18q22.1q23 region, and its clinical manifestations included microcephaly and comprehensive developmental disorder in addition to DRE [27]. Reportedly, copy number variation (CNV) constitutes approximately 10% of hereditary epilepsy cases [28]. An international study has illuminated the transformative potential of a definitive genetic diagnosis in the realm of clinical management [29]. It has been unveiled that when clinical management was tailored in response to genetic testing outcomes, approximately 64.7% of patients witnessed a reduction or even elimination of seizures.This underscores the profound influence of genetic testing on enhancing patient outcomes among individuals grappling with epilepsy [29].

The significance of immunological factors in DRE should not be disregarded. In 2020, the ILAE introduced the term “autoimmune epilepsy” denoting the persistence of seizures despite appropriate immunotherapy without substantial evidence of inflammatory activity [30]. This condition can manifest in patients displaying high titers of GAD65 antibodies, tumor-related antibodies, and Rasmussen’s encephalitis. It also encompasses a small subset of patients who continue to experience seizures even after the acute phase of autoimmune encephalitis has passed. Unlike acute symptomatic seizures secondary to autoimmune encephalitis, autoimmune-related epilepsy manifests as drug-resistant. Even after adequate immunotherapy for the primary condition and standardized anti-seizure treatment, seizures persist chronically and long-term, proving challenging to control solely through medications. Research suggests that neuronal apoptosis induced by T cell cytotoxicity stands as the central pathological mechanism, potentially resulting in structural damage to the cerebral hemisphere. The presence of antibodies might represent a consequential byproduct in this pathological process, hinting that comprehensive immunotherapy might not effectively diminish seizure frequency [31]. Therefore, clinicians are encouraged to consider timely immune-targeted therapy, symptomatic supportive treatment, and actively explore opportunities for surgical intervention to potentially enhance disease prognosis.

Within the scope of this study, twelve children exhibited a dual etiology involving genetic factors along with structural or metabolic elements. These etiological factors may be either causally related or act independently. Among them, four cases presented with tuberous sclerosis (TSC) evident on MRI scans. Three cases were found to harbor variants in the TSC1 gene, while one case exhibited variants in the TSC2 gene. These gene variants are situated within the mTOR pathway and are known to trigger excessive activation of the mTOR pathway. This, in turn, disrupts normal cell proliferation and ultimately contributes to the development of malformed cerebral cortex, a condition closely associated with the onset of DRE [32]. Furthermore, three cases exhibited epileptic seizures attributable to mitochondrial gene variants (POLG, MT-TL1, DNM1L), as documented in previous studies [33–35]. Mitochondria-associated epilepsy entails a multifaceted pathogenesis, giving rise to various seizure types, predominantly drug-resistant, coupled with multisystem involvement and an unfavorable prognosis [36]. When faced with DRE of indeterminate etiology in a clinical setting, it is imperative to consider the potential involvement of mitochondrial diseases.

In this study, 46.7% of cases had an undetermined etiology, with 48.7% (38 out of 78) attributed to incomplete genetic testing, without excluding the presence of a certain proportion of genetic factors. For children with DRE where the cause is unclear or there is no surgical indication, alternative non-pharmacological therapies can be employed in anti-seizure treatment. Ketogenic Diet (KD), as a traditional non-pharmacological intervention, constitutes a dietary formula characterized by a high proportion of fats, low carbohydrates, and appropriate levels of proteins and other nutrients. By restricting the intake of dietary fiber carbohydrates, thereby emulating the metabolic state of the body under conditions of hunger, it can effectively diminish the frequency and severity of epileptic seizures. KD is applicable to children across various age groups experiencing frequent seizures associated with DRE and metabolic disorders such as Glucose Transporter 1 Deficiency Syndrome (GLUT-1). The therapeutic outcomes demonstrate a reduction of seizure frequency by over 50% in 50–80% of refractory epilepsy cases, achieving complete seizure freedom in 10–20% of cases, and enhancing overall quality of life by improving cognition, behavior, and sleep quality [6]. Another rapidly advancing non-pharmacological therapy is neuromodulation, primarily encompassing Vagus Nerve Stimulation (VNS), Responsive Neurostimulation (RNS), and Deep Brain Stimulation (DBS) [7]. Neuromodulation is applicable to patients with poorly controlled epilepsy who are not suitable candidates for resective surgery. According to research findings [7], the average improvement rate of DRE with VNS therapy is approximately 34.7%. For DBS, there is an average reduction of 41% in seizure frequency after one year. After five years post-surgery, 68% of patients experience at least a 50% reduction in seizure frequency, with 16% of patients achieving seizure-free periods exceeding six months [37]. Regarding RNS, it can completely control about 10–15% of refractory epilepsy seizures [38]. Therefore, it is considered that neuromodulation stands as one of the effective therapeutic choices for DRE. However, each treatment modality possesses its unique advantages and limitations, requiring personalized assessments before formulating a plan.

There are certain limitations in our study to acknowledge: (1)This study is a single-center endeavor, characterized by a relatively modest sample size, potentially introducing a degree of bias in the distribution of etiological factors. (2)The retrospective nature of this study restricts its exploration of etiological factors to a cross-sectional approach. It is thus advisable to embark on a prospective cohort study as the subsequent course of action, allowing for the monitoring of seizures in children with DRE following adjustments in their treatment regimens in accordance with the identified etiology.

## Conclusions

DRE in children has a complicated aetiology, predominantly characterized by structural and genetic factors. Onset ages vary among different causative factors. The early onset of genetic causes emphasizes the significance of genetic testing in children presenting with seizures at an early age. This study gives guidance for the etiology distribution of DRE in children as well as a foundation for treatment selection. Early improvement of the etiological examination and early diagnosis of DRE is not only conducive to patients and their families accepting relevant knowledge and preparing for standardized long-term treatment, but it is also conducive to epilepsy specialists considering various treatment methods other than drug therapy to improve patients’ prognosis.

### Electronic supplementary material

Below is the link to the electronic supplementary material.

Supplementary Material 1

Supplementary Material 2

## Data Availability

The data that support the findings of this study are available on request from the corresponding author.The data are not publicly available due to privacy or ethical restrictions.

## Abbreviations

DRE: Drug-refractory epilepsy
ASMS: Antiseizure medications
ILAE: International League against Epilepsy
VEEG: Video electroencephalogram. IESS:Infantile epileptic spasm syndrome
EIDEE: Early-infantile developmental and/or epileptic encephalopathies
DEES: Developmental and/or epileptic encephalopathies
SeLFEs: Self-limited focal epilepsies
MCD: Malformations of cortical development
TSC: Tuberous sclerosis complex
KD: Ketogenic Diet
GLUT-1: Glucose Transporter 1 Deficiency Syndrome
VNS: Vagus Nerve Stimulation
RNS: Responsive Neurostimulation
DBS: Deep Brain Stimulation

## References

[1] Fisher RS, van Emde BW, Blume W, Elger C, Genton P, Lee P (2005). Epileptic seizures and epilepsy: definitions proposed by the international league against epilepsy (ilae) and the international bureau for epilepsy (ibe). Epilepsia.

[2] Kalilani L, Sun X, Pelgrims B, Noack-Rink M, Villanueva V (2018). The epidemiology of drug-resistant epilepsy: a systematic review and meta-analysis. Epilepsia.

[3] World healthorganization. Epilepsy: a public healthimperative. World health organization; 2019.

[4] Janson MT, Bainbridge JL (2021). Continuing burden of refractory epilepsy. Ann Pharmacother.

[5] Chen Z, Brodie MJ, Liew D, Kwan P (2018). Treatment outcomes in patients with newly diagnosed epilepsy treated with established and new antiepileptic drugs: a 30-year longitudinal cohort study. Jama Neurol.

[6] Ulamek-Koziol M, Czuczwar SJ, Januszewski S, Pluta R. Ketogenic diet and epilepsy. Nutrients. 2019;11(10). 10.3390/nu11102510. 10.3390/nu11102510 PMC6836058 31635247

[7] Touma L, Dansereau B, Chan AY, Jette N, Kwon CS, Braun K (2022). Neurostimulation in people with drug-resistant epilepsy: systematic review and meta-analysis from the ilae surgical therapies commission. Epilepsia.

[8] Dwivedi R, Ramanujam B, Chandra PS, Sapra S, Gulati S, Kalaivani M (2017). Surgery for drug-resistant epilepsy in children. N Engl J Med.

[9] Kwan P, Arzimanoglou A, Berg AT, Brodie MJ, Allen HW, Mathern G (2010). Definition of drug resistant epilepsy: consensus proposal by the ad hoc task force of the ilae commission on therapeutic strategies. Epilepsia.

[10] Wirrell EC, Nabbout R, Scheffer IE, Alsaadi T, Bogacz A, French JA (2022). Methodology for classification and definition of epilepsy syndromes with list of syndromes: report of the ilae task force on nosology and definitions. Epilepsia.

[11] Fisher RS, Cross JH, French JA, Higurashi N, Hirsch E, Jansen FE (2017). Operational classification of seizure types by the international league against epilepsy: position paper of the ilae commission for classification and terminology. Epilepsia.

[12] Richards S, Aziz N, Bale S, Bick D, Das S, Gastier-Foster J (2015). Standards and guidelines for the interpretation of sequence variants: a joint consensus recommendation of the American college of medical genetics and genomics and the association for molecular pathology. Genet Med.

[13] Scheffer IE, Berkovic S, Capovilla G, Connolly MB, French J, Guilhoto L (2017). Ilae classification of the epilepsies: position paper of the ilae commission for classification and terminology. Epilepsia.

[14] Pérez-Pérez D, Frías-Soria CL, Rocha L (2021). Drug-resistant epilepsy: from multiple hypotheses to an integral explanation using preclinical resources. Epilepsy Behav.

[15] Vitaliti G, Pavone P, Marino S, Saporito M, Corsello G, Falsaperla R (2019). Molecular mechanism involved in the pathogenesis of early-onset epileptic encephalopathy. Front Molec Neurosci.

[16] Perucca E, Perucca P, White HS, Wirrell EC (2023). Drug resistance in epilepsy. Lancet Neurol.

[17] Costagliola G, Depietri G, Michev A, Riva A, Foiadelli T, Savasta S (2022). Targeting inflammatory mediators in epilepsy: a systematic review of its molecular basis and clinical applications. Front Neurol.

[18] Jehi L, Jette N, Kwon CS, Josephson CB, Burneo JG, Cendes F (2022). Timing of referral to evaluate for epilepsy surgery: expert consensus recommendations from the surgical therapies commission of the international league against epilepsy. Epilepsia.

[19] Song TY, Deng J, Fang F, Chen CH, Wang XH, Wang X (2021). The etiology of 340 infants with early-onset epilepsy. Chin J Pediatr.

[20] Pillai SC, Mohammad SS, Hacohen Y, Tantsis E, Prelog K, Barnes EH (2016). Postencephalitic epilepsy and drug-resistant epilepsy after infectious and antibody-associated encephalitis in childhood: clinical and etiologic risk factors. Epilepsia.

[21] Hale AT, Chari A, Scott RC, Helen CJ, Rozzelle CJ, Blount JP et al. Expedited epilepsy surgery prior to drug resistance in children: a frontier worth crossing? Brain 2022, 145(11):3755–62.10.1093/brain/awac275. 10.1093/brain/awac275 35883201

[22] Guery D, Rheims S (2021). Clinical management of drug resistant epilepsy: a review on current strategies. Neuropsychiatr Dis Treat.

[23] He Z, Li Y, Zhao X, Li B (2022). Dravet syndrome: advances in etiology, clinical presentation, and treatment. Epilepsy Res.

[24] Pavone P, Falsaperla R, Ruggieri M, Pratico AD, Pavone L (2013). West syndrome treatment: new roads for an old syndrome. Front Neurol.

[25] Cardenas-Rodriguez N, Carmona-Aparicio L, Perez-Lozano DL, Ortega-Cuellar D, Gomez-Manzo S, Ignacio-Mejia I (2020). Genetic variations associated with pharmacoresistant epilepsy (review). Mol Med Rep.

[26] Liu J, Tong L, Song S, Niu Y, Li J, Wu X (2018). Novel and de novo mutations in pediatric refractory epilepsy. Mol Brain.

[27] Verrotti A, Carelli A, di Genova L, Striano P (2015). Epilepsy and chromosome 18 abnormalities: a review. Seizure.

[28] Coppola A, Cellini E, Stamberger H, Saarentaus E, Cetica V, Lal D (2019). Diagnostic implications of genetic copy number variation in epilepsy plus. Epilepsia.

[29] McKnight D, Morales A, Hatchell KE, Bristow SL, Bonkowsky JL, Perry MS (2022). Genetic testing to inform epilepsy treatment management from an international study of clinical practice. Jama Neurol.

[30] Steriade C, Britton J, Dale RC, Gadoth A, Irani SR, Linnoila J (2020). Acute symptomatic seizures secondary to autoimmune encephalitis and autoimmune-associated epilepsy: conceptual definitions. Epilepsia.

[31] Bien CG, Vincent A, Barnett MH, Becker AJ, Blumcke I, Graus F (2012). Immunopathology of autoantibody-associated encephalitides: clues for pathogenesis. Brain.

[32] Li Y, Si Z, Zhao W, Xie C, Zhang X, Liu J (2023). Tuberous sclerosis complex: a case report and literature review. Ital J Pediatr.

[33] Rahman S, Copeland WC (2019). Polg-related disorders and their neurological manifestations. Nat Rev Neurol.

[34] Tetsuka S, Ogawa T, Hashimoto R, Kato H (2021). Clinical features, pathogenesis, and management of stroke-like episodes due to melas. Metab Brain Dis.

[35] Ryan CS, Fine AL, Cohen AL, Schiltz BM, Renaud DL, Wirrell EC (2018). De novo dnm1l variant in a teenager with progressive paroxysmal dystonia and lethal super-refractory myoclonic status epilepticus. J Child Neurol.

[36] Lopriore P, Gomes F, Montano V, Siciliano G, Mancuso M. Mitochondrial epilepsy, a challenge for neurologists. Int J Mol Sci. 2022;23(21). 10.3390/ijms232113216. 10.3390/ijms232113216 PMC9656379 36362003

[37] Salanova V, Witt T, Worth R, Henry TR, Gross RE, Nazzaro JM (2015). Long-term efficacy and safety of thalamic stimulation for drug-resistant partial epilepsy. Neurology.

[38] Inaji M, Yamamoto T, Kawai K, Maehara T, Doyle WK (2021). Responsive neurostimulation as a novel palliative option in epilepsy surgery. Neurol Med -Chir.

