# Supplementary material for: Etiological analysis of 167 cases of drug-resistant epilepsy in children
Source: Ital J Pediatr. 2024 Mar 13;50:50. doi: 10.1186/s13052-024-01619-8 (PMC10938754; doi:10.1186/s13052-024-01619-8)
Supplement: Supplementary file 2 — Supplementary Material 2 [file 13052_2024_1619_MOESM2_ESM.docx]

Dear Editors:

We would like to submit the enclosed manuscript entitled “Etiological analysis of 167 cases of drug-resistant epilepsy in children”, which we wish to be considered for publication in “Italian Journal of Pediatrics”. No conflict of interest exits in the submission of this manuscript, and this manuscript is approved by all authors for publication. I would like to declare on behalf of my co-authors that the work described was original research that has not been published previously, and not under consideration for publication elsewhere, in whole or in part. All the authors listed have approved the manuscript that is enclosed.

In this work, we summarized and analyzed the etiological distribution, age distribution and clinical manifestations of 167 children with drug-resistant epilepsy, which can improve the clinical understanding of children with DRE. In addition, after statistical analysis of the age of onset of each etiology, it was found that the age of onset of genetic etiology was earlier, so genetic testing should be improved as soon as possible for DRE children with unknown causes. This study provides valuable guidance for the early diagnosis of DRE in the clinic, and lays a foundation for the formulation of treatment plans for children with DRE. So I think this paper is suitable for “Italian Journal of Pediatrics”.

We deeply appreciate your consideration of our manuscript, and we look forward to receiving comments from the reviewers. If you have any queries, please don’t hesitate to contact me at the address below.

Thank you and best regards.

Yours sincerely,

Ranran Zuo

Corresponding author:

Name: Suzhen Sun

E-mail: sunsuzhen2004@126.com
