# Supplementary material for: Etiological analysis of 167 cases of drug-resistant epilepsy in children
Source: Ital J Pediatr. 2024 Mar 13;50:50. doi: 10.1186/s13052-024-01619-8 (PMC10938754; doi:10.1186/s13052-024-01619-8)
Supplement: Supplementary file 1 — Supplementary Material 1 [file 13052_2024_1619_MOESM1_ESM.docx]

Dear Dr. Raffaele Falsaperla and Reviewers:

Thank you very much for your consideration and for the reviewers’ comments on our manuscript entitled “Etiological analysis of 167 cases of drug-resistant epilepsy in children”. We are very grateful to receive your comments which are very helpful for improving our paper. We have tried our best to improve the manuscript and made some changes that did not affect the content and framework of the paper. Detailed point-by-point response is given below. The reviewer comments are laid out below in bold font and specific concerns have been numbered. Our response is given in blue and changes/additions to the manuscript are given in the blue text.

I hope that the revised manuscript is suitable for publication in Italian Journal of Pediatrics. Thank you again for your help with the manuscript. I am more than happy to provide any further information you may need.

Yours sincerely,

Ran-Ran Zuo

Corresponding author:

Name: Su-Zhen Sun

E-mail: [sunsuzhen2004@126.com](mailto:sunsuzhen2004@126.com)

Responses to Reviewer #1:

**I congratulate you for the work you have done and especially for the topic you have chosen, which is of considerable interest not only to the pediatric neurologist but to all professionals who care for children with drug-resistant epilepsy.**

**- Well the abstract which is brief and concise; the objective of the work and its clinical utility are well stated and clear**

Reply: Thank you for your positive comments; it has provided me with immense encouragement. We have carefully revised the whole manuscript according to your comments.

**Q1.In the introduction I appreciated the mention regarding psychiatric comorbidities; I recommend dwelling a little more on the pharmacological and nonpharmacological treatment of drug-resistant epilepsy**

Reply: Thanks for your comments. We have appended the pharmacological and non-pharmacological treatment landscape of DRE in the introduction.（See at line 61-69）

**Q2.In the methods I recommend including a specific section for limitations so as to make the reading more fluent and schematic.**

Reply: We agree with the reviewer. We added several paragraphs to the method with the aim of making the process of the study more rigorous. These paragraphs include diagnostic criteria for DRE, diagnostic criteria for epilepsy syndrome, assessment of seizure type, and detailed records of auxiliary examinations employed during the research process.（See at line 87-102, 115-119）

**Q3.It might be useful to include hints about the type of genetic tests recommended, given the considerable etiologic and therapeutic importance involved; this information could be beneficial especially to pediatricians of free choice or cmq non-experts in pediatric neurology.**

Reply: Thanks for your valuable suggestion. Due to the limited number of genes covered by Panel sequencing and the need to constantly update the genes included in the test package, we employed familial whole-exome sequencing for genetic variant screening. Based on the clinical presentations of the patients, some children were added to the chromosome karyotype analysis, copy number variation screening and mitochondrial gene testing.（See at line 108-114）

**Q4.Discussion: I suggest increasing the bibliography. I suggest this interesting work on west syndrome: Pavone P, Falsaperla R, Ruggieri M, Praticò AD, Pavone L. West syndrome treatment: new roads for an old syndrome. Front Neurol. 2013 Aug 12;4:113. doi: 10.3389/fneur.2013.00113. PMID: 23964265; PMCID: PMC3740275.**

Reply: We sincerely appreciate the valuable comments. West Syndrome is encompassed within the Infantile Spasms Syndrome(IESS), typically associated with genetic factors, and the majority of cases have an unfavorable prognosis. We have added this bibliography to the discussion, as suggested by the reviewers.(See at line 277-278)

**Q5.I suggest also including mentions of nonpharmacological treatment methods such as ketogenic therapy. I noticed only one mention of alternative therapies and relegated to the conclusion.**

Reply: Thanks for your comments. We am sorry that non-pharmacological therapies were not described in the first draft, and we added to the discussion other methods besides drugs and surgical treatment, such as the indications of ketogenic diet and neurostimulation in the treatment of DRE and efficacy. (See at line 329-357)

**Q6.doi missing in bibliography**

Reply: Thanks for your suggestion. We have added DOI numbers to the bibliography.

Responses to Reviewer #2:

**Thanks for inviting to review manuscript Etiological analysis of 167 cases of drug-resistant epilepsy (DRE) in children. The Authors report the etiological distribution characteristics of drug-resistant epilepsy (DRE) in children, with the aim of providing valuable perspectives to enhance clinical practice.**

**Q1.Being this a retrospective study the Methods must be more rigorous and for this the study adhered to the Strengthening the Reporting of Observational Studies in Epidemiology (STROBE) checklist for reporting.**

Reply: Thanks for your valuable advice. We have carefully read and checked the contents of the list of Strengthening epidemiological Observational Studies reports (STROBE), made some minor adjustments to the structure of the article, and included potential biases in the limitations section.

**Q2.Please the Authorsshoul create a paragraph on limitations and one on what the paper add to the current literarure.**

Reply: Thanks for your professional suggestion. We describe the limitations present in the study.(See at line 358-365). In the first paragraph of the discussion, we provided a description of the study results and their significance. The analysis of etiology contributes to the advancement of precise diagnosis and treatment of DRE.(See at line 228-238).

**Q3.The Discussion section should have a premise on mechanism in the pathogenesis of DRE ( see Molecular Mechanism Involved in the Pathogenesis of Early-Onset Epileptic Encephalopathy. Vitaliti G, Front Mol Neurosci. 2019 May 15;12:118)**

Reply: We sincerely appreciate the valuable comments. Through literature review, we have summarized the potential pathogenic mechanisms of DRE. However, these six main hypotheses each have their limitations and overlapping aspects. Recent research has emphasized the role of neuroinflammation in the development of DRE and prolonged seizures. Some anti-cytokine drugs, such as IL-1, IL-6, and CD20, have shown considerable efficacy in prolonged seizures. It is hoped that future studies will explore this mechanism further, advancing our understanding of the development of DRE and prolonged seizures.(See at line 239-255)
